# A randomized controlled trial of a skills training for oncologists and a communication aid for patients to stimulate shared decision making about palliative systemic treatment (CHOICE): study protocol

**DOI:** 10.1186/s12885-017-3838-8

**Published:** 2018-01-08

**Authors:** I. Henselmans, E. M. A. Smets, J. C. J. M. de Haes, M. G. W. Dijkgraaf, F. Y. de Vos, H. W. M. van Laarhoven

**Affiliations:** 1 Department of Medical Psychology, Academic Medical Center, University of Amsterdam, PO Box 22660, 1100 DD Amsterdam, the Netherlands; 2 Clinical Research Unit, Academic Medical Center, University of Amsterdam, Amsterdam, the Netherlands; 3 Department of Medical Oncology, University Medical Center Utrecht, Utrecht, The Netherlands; 4 Department of Medical Oncology, Academic Medical Center, University of Amsterdam, Amsterdam, the Netherlands

**Keywords:** Shared decision making, Advanced cancer, Palliative medicine, Systemic treatment, Doctor-patient communication, Patient participation, Patient education, Skills training

## Abstract

**Background:**

Systemic treatment for advanced cancer offers uncertain and sometimes little benefit while the burden can be high. Hence, treatment decisions require Shared Decision Making (SDM). The CHOICE trial examines the separate and combined effect of oncologist training and a patient communication aid on SDM in consultations about palliative systemic treatment.

**Methods:**

A RCT design with four parallel arms will be adopted. Patients with metastatic or irresectable cancer with a median life expectancy <12 months who meet with a medical oncologist to discuss the start or continuation of palliative systemic treatment are eligible. A total of 24 oncologists (in training) and 192 patients will be recruited. The oncologist training consists of a reader, two group sessions (3.5 h; including modelling videos and role play), a booster feedback session (1 h) and a consultation room tool. The patient communication aid consists of a home-sent question prompt list and a value clarification exercise to prepare patients for SDM in the consultation. The control condition consists of care as usual. The primary outcome is observed SDM in audio-recorded consultations. Secondary outcomes include patient and oncologist evaluation of communication and decision-making, the decision made, quality of life, potential adverse outcomes such as anxiety and hopelessness, and consultation duration. Patients fill out questionnaires at baseline (T0), before (T1) and after the consultation (T2) and at 3 and 6 months (T3 and T4). All oncologists participate in two standardized patient assessments (before-after training) prior to the start of patient inclusion. They will fill out a questionnaire before and after these assessments, as well as after each of the recorded consultations in clinical practice.

**Discussion:**

The CHOICE trial will enable evidence-based choices regarding the investment in SDM interventions targeting either oncologists, patients or both in the advanced cancer setting. The trial takes into account the immediate effect of the interventions on observed communication, but also on more distal and potential adverse patient outcomes. Also, the trial provides evidence regarding the assumption that SDM about palliative cancer treatment results in less aggressive treatment and more quality of life in the final period of life.

**Trial registration:**

Netherlands Trial Registry number NTR5489 (prospective; 15 Sep 2015).

**Electronic supplementary material:**

The online version of this article (10.1186/s12885-017-3838-8) contains supplementary material, which is available to authorized users.

## Background

World-wide, 8.2 million people die of cancer each year [1]. In the final period of life, disease-targeted treatment offers uncertain and sometimes little benefit while the burden of treatment can be high. Hence, treatment decisions are highly preference-sensitive [2]. Decision making cannot be based solely on evidence and patients’ clinical status, but should incorporate patients’ values and preferences [3]. This requires shared decision making (SDM), an approach whereby clinician and patient exchange information and jointly deliberate to come to an agreed-upon decision [4–6]. SDM is increasingly advocated because of the ethical imperative to involve patients [7] as well as the increasing evidence for beneficial patient outcomes, such as satisfaction with communication and well-being [8, 9]. Moreover, yet tentatively, SDM in the final period of life might result in more attention to symptom control and less (aggressive) medical interventions [10–12]. For example, in the curative setting, the use of patient decision aids resulted in reduced choice of invasive surgery and screening [13].

### Decision making in advanced cancer

SDM implies that clinicians provide accurate and balanced information about the available treatment options and invite patients to express their values and preferences [14]. In consultations with advanced cancer patients, such a two-way flow of information is not always achieved. Oncologists’ information provision is often incomplete, e.g., oncologists infrequently discuss the expected survival benefits of palliative systemic treatment or the option to refrain from systemic treatment [15–17] and communication about prognosis and death is often implicit [18, 19]. Moreover, a recent study demonstrated that joint elaboration and preference construction is not standard practice [20], particularly once systemic treatment has started.^.^ In sum, systemic treatment for advanced cancer may be prescribed and continued without conscious and joint deliberation.

Several factors make SDM particularly challenging in consultations with advanced cancer patients. SDM requires high-level communication skills known to be demanding for clinicians, such as tailoring information provision to the individual patients’ information needs [21, 22], dealing with patients’ emotions in response to bad news [23] and coaching patients in constructing a treatment preference. Furthermore, SDM puts patients in an active and participatory role. Given the complexity and significance of the topics discussed in consultations about treatment for advanced cancer, as well as the highly vulnerable and dependent position of patients, this may be challenging [24]. Lastly, SDM can be particularly sensitive in this context as it deals with the imminent end of life. Both oncologists and patients have been shown to prefer to keep a focus on the short term and on ‘beating’ the cancer, instead of anticipating what is to come [25–27]. Such focus may enable the patient to retain a sense of hope, even if misguided, and an often desired ‘fighting spirit’. At the same time, it may prevent decisions in keeping with the patient’s preferences.

### Training oncologists

Commonly, the performance of providers is addressed to enhance communication in medical encounters [28–30]. It has been shown that training significantly improves the observed communication skills in simulated settings as well as in clinical practice on the short term [31–34]. However, there is much less evidence for the consolidation of this effect on the long-term and for an effect on more distal patient outcomes. Reviews demonstrated that training programs specifically focused on SDM vary widely in format and components with little robust evidence for efficacy [35–37]. Common components are role play, discussion, education in small groups, printed materials, and audit/feedback [35]. Effective means are small trainee groups; an experienced trainer and booster sessions to refresh training content [33, 38]. The evidence for a dose-response relationship is not conclusive [32, 38], although it has been suggested that training for minimally 3 days is more likely to result in behavior change [33]. To our knowledge, no training program has explicitly focused on teaching SDM about palliative cancer treatment. The current trial examines the effect of a training that aims to increase SDM regarding palliative systemic treatment through addressing medical oncologists’ knowledge, beliefs and skills. It combines printed material, video modelling, education, small groups discussion, role play, and a booster session.

### Supporting patients

Besides training providers, there is growing evidence for patient-targeted communication support to improve communication. Interventions targeting patients were shown to enhance patient participation in oncology consultations in general [39], as well as in the palliative setting [30, 40]. Moreover, there is evidence suggesting that preparing patients can improve SDM in consultations [41], although the effect may be smaller than the effect of provider-targeted interventions [37]. A well-known and widely implemented tool to support patients in decision making is a decision aid (DA). A DA usually presents treatment options as well as the pros and cons of the options in paper or digital format, and offers value clarification exercises that help patients formulating a treatment preference [42, 43]. A DA can be used independently by patients, but use of the aid in or next to a conversation with a care provider is recommended [44, 45]. DA’s have been developed for the advanced cancer setting [46–48]. These studies report on a positive effect on patients’ understanding, but not on the decision made, patients’ decisional role and post-decision confidence. Another well-known patient-targeted communication tool is a Question Prompt List (QPL), a structured booklet with example questions patients can ask their doctor. A QPL is shown to facilitate cancer patients’ participation in consultations [49]. It stimulates agenda setting, assists patients in formulating and remembering important questions, and helps to put difficult issues on the agenda. Moreover, a QPL gives patients control over the oncologist’s information provision and enhances information giving about topics oncologists regularly fail to address, such as prognosis [50]. A QPL focused on end-of-life issues for patients with incurable cancer proved to be an acceptable tool in consultations with a medical oncologist [51–53].

The current study examines the effect of a communication aid focused on SDM about palliative systemic treatment that combines elements from both DA’s (value clarification exercises) and QPL’s (question prompts). It can be used flexibly regardless of the type and number of available treatment options, and explicitly targets the two-way flow of information within a conversation between patient and oncologist.

### Targeting both parties

Leaving out one party in a communication intervention might be similar to ‘anticipating an elegant waltz to emerge on the ballroom when only one partner has taken dance lessons’ [54]. Indeed, interventions to improve communication may be most effective if they target both doctors and patients [30, 37]. Focusing only on patients and not on doctors might even have unintended negative effects [55–57]. For instance, Australian breast cancer patients who received a preparation package (including a QPL) participated more actively in the consultation than women who did not receive such a package. However, they were less likely to reach their preferred level of involvement in the consultation [56]. Similarly, another Australian study showed that providing cancer patients with a QPL which was not actively endorsed by the oncologist, did result in more observed patient participation, but also in longer consultations and more patient anxiety post-consultation [58]. Recently, it was shown that the combination of a skills training for oncologists and a coaching session including a QPL for advanced cancer patients improves patient-centered communication, yet not secondary outcomes such as quality of life and care received at the end of life [59, 60]. To the best of our knowledge, no studies have examined the independent and combined effect of a patient- and an oncologist-targeted intervention on SDM in consultations with advanced cancer patients.

### Aims

The aim of the CHOICE trial (CHOosing treatment together In Cancer at the End of life) is to evaluate the effectiveness of a patient communication aid and an oncologist training on shared decision making regarding palliative systemic treatment for cancer patients with an estimated life expectancy of <1 year. The *primary outcome* is observed SDM in decision-making consultations. We hypothesize that each of the interventions will separately improve observed SDM and that the combination of both will be more effective in improving observed SDM than targeting only one party.

For secondary outcomes, we hypothesize a positive effect of the interventions on immediate outcomes related to communication and decision making. We expect the oncologist training to improve observed SDM in standardized patient assessments as well as observed general communication skills in standardized and in actual encounters. Furthermore, we hypothesize a positive effect of the interventions on patients’ perceived efficacy, satisfaction, perceived empathy, achievement of the preferred role in decision making, perceived shared decision making, evaluation of the decision made and patients’ trust in the oncologist and oncologists’ post-consultation satisfaction. Moreover, we hypothesize an effect of the interventions on *distal outcomes*, i.e., the decision made, patients’ attitude towards length or quality of life, and patients’ quality of life. We will also explore the effect of the interventions on *potentially adverse outcomes*, i.e., patients’ anxiety, loss of fighting spirit and hope. Lastly, we will explore the effect of the interventions on consultation duration and examine patients’ use and evaluation of the communication aid.

## Methods/design

This protocol is written in accordance with the CONSORT statement for reporting parallel group randomized trials [61].

### Design

The study adopts a randomized controlled trial design with four parallel arms. Medical oncologists (in training) will be randomized to receive the training or to continue their standard practice. Patients (within oncologists) will be randomized to either receive the communication aid or care as usual.

### Study setting

The study will be conducted on medical oncology departments of both academic and non-academic hospitals in the Netherlands.

### Participants

The source population consists of (1) all patients with metastatic or inoperable tumors for whom survival curves indicate a median life expectancy of <12 months without disease targeted treatment, for whom systemic palliative treatment does not offer median survival benefit of >6 months (at group-level), who meet with the oncologist to discuss either the start of (a new line) of treatment or the (dis)continuation or adjustment of current treatment, and (2) medical oncologists as well as medical oncologists in training who communicate with these patients in decision-making consultations. Eligible are all oncologists (in-training) who are qualified to communicate with patients independently. Oncologists involved in the design of the interventions will be excluded. Eligibility criteria for patients are presented in Table 1. For some patients, eligibility for systemic treatment (yes, no or unsure) could only be assessed during the consultation (exclusion criterion 5 and 6). As a result, some patients will be judged non-eligible and will be excluded post-hoc (i.e., after randomization and first data collection).

**Table 1:** Patient eligibility criteria

*Patient inclusion criteria*	
1. patients diagnosed with metastatic or locally irresectable cancer 2. not eligible for treatment with curative intent 3. a median life expectancy of < 1 year without systemic treatment, and a median survival benefit of systemic treatment of < 6 months, which includes, but is not limited to: a. patients with metastases or locally irresectable tumors of the pancreas, esophagus, stomach, liver, (gall) bladder, and patients with metastatic sarcoma or melanoma b. patients with advanced cancer, irrespective of tumor type, who have experienced progression under first line palliative systemic treatment. 4. scheduled for a consultation with a participating medical oncologist (in training) in which decisions about the start, (dis)continuation or adjustment of palliative systemic treatment will be made. This includes: *a. initial consultations* in which a decision to start, forego or postpone a (new line of) systemic treatment will be made *b. evaluative consultations* in which current treatment is evaluated, usually after a fixed number of cycles (with a CT/PET-CT) and a decision to (dis)continue and/or adjust systemic treatment will be made	
*Patient exclusion criteria*	
1. patients who have insufficient mastery of Dutch to understand the communication aid and questionnaires as judged by either the physician or the researcher 2. cognitive disabilities or a psychiatric disorder that hinder understanding of the communication aid and questionnaires as judged by either the physician or the researcher 3. insufficient time between identification and consultation (< 2 working days) 4. a primary brain tumor or brain metastasizes which significantly hinder cognitive functioning 5. not/no longer/not yet eligible for (an additional line of) palliative systemic treatment (standard or experimental), which prevented discussion of systemic treatment as judged by the physician 6. insufficient diagnostic information available, which prevented discussion of systemic treatment as judged by the physician	

### Sample size

The trial is powered to detect an independent effect of the two interventions on observed SDM, as assessed with the OPTION12 [62, 63], a widely used instrument to assess SDM in consultations. There is no agreement on what constitutes a clinically meaningful difference on the OPTION12. The sizes of the effects of SDM interventions on OPTION12, including decision aids and physician training across different specialties and in both simulated and clinical settings, range from 0.5 to 1.5 [41, 64, 65]. As most reported large effect sizes, we adopted an effect size of d = 0.8 in the sample size calculation. We expected that the effect of the combination of the oncologist training and the patient communication aid will be larger, but not twice as large (estimated effect size = 1.2). To account for this hypothesized antagonistic interaction [66], we decided to adopt a parallel group design instead of a factorial design. Hence, we will examine the effect of the oncologist training among patients allocated to the control condition (between-oncologist comparison) and the effect of the patient communication aid among untrained oncologists (within-oncologist comparison).

SDM will vary by oncologist. ICC’s for the OPTION12 reported in literature [67, 68] and personal communication (Kunneman, Leiden University, the Netherland, May 2014; Kriston, University Medical Center Hamburg, Germany, July 2014) range from 0.11 to 0.60. In a sample of 47 audio-recorded consultations meeting the same eligibility criteria as the current trial [20], an ICC of 0.19 for OPTION12 scores was demonstrated (unpublished). Since the ICC was 0.20 or lower in the majority of these data sets, a value of 0.20 was adopted in the sample size calculation.

Sample size calculation was based on the effect of the oncologist training (between-oncologist comparison; two-sided t-test taking into account clustered data). The above mentioned criteria, along with a power of >80% and α of 0.05, resulted in a required sample size of 24 oncologists and 192 patients (12 oncologists per arm and 8 patients per oncologist; power = 0.84).

### Recruitment

#### Oncologists

To include 24 oncologists (in training), the medical oncology departments of both academic and non-academic hospitals are approached through existing networks until at least 30 eligible and consenting oncologists are recruited (considering potential drop-out of 25%). Oncologists are informed about the study by both the local investigator and the principal investigator; are provided with an information letter and are asked for written informed consent.

#### Patients

In a previous observational study on doctor-patient communication, the response rate was 78%. We more conservatively estimated the response rate to be 66% for this RCT, implying that *n* = 291 patients need to be approached to include *n* = 192 patients. Roles and procedures for inclusion differ per center. Eligible patients are identified from the outpatient clinic agendas at least one week in advance by the oncologist (in the coordinating hospital supported by the research team), or by a designated oncologist or nurse. Depending by center, either the treating oncologist, a nurse or trial officer contacts the patient by telephone, explains the study in general terms and asks for permission to provide the patient’s contact details (along with gender, tumor type, treating oncologist, date of consultation and type of consultation) to the coordinating research team. The coordinating research team then informs the patient by telephone about the study in more detail. Patients are told that the aim of the study is to investigate the effect of an oncologist training and a patient communication aid on doctor-patient communication about treatment. Since time between initial contact and consultation is often short, patients will be randomized immediately after providing oral informed consent. Patients will subsequently receive a written information letter with information about the study and a written informed consent form. This package will include information about the condition they were assigned to (and, depending on condition, the communication aid). Patients are requested to bring the consent forms to the consultation, where they will sign the forms together with either the oncologist or a research associate. Patients will be given the opportunity to ask questions. Companions are also asked to provided written consent for audio-recording the consultation. It is stressed that patients can withdraw consent at any time. In all stages, reasons to decline participation or drop out will be documented.

### Procedure and time line

**Fig. 1:**
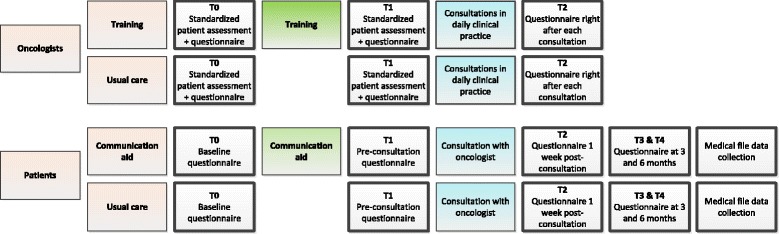
Assessments for oncologists and patients

Figure 1 shows the time line for oncologists and patients. *Oncologists* will participate in a simulated encounter with an actor patient (standardized patient assessment) at two points in time, with 2–3 months in between. After each assessment they will complete a questionnaire (T0 and T1). Oncologists randomized to the training condition will receive the skills training between the first and second standardized patient assessment. Subsequently, for each oncologist, we aim to audio-record consultations with 8 patients in daily clinical practice with a small audio-recording device. Either a research associate or the oncologist will operate the recorder. The research associate will not attend the consultation. After each consultation, oncologists complete a short questionnaire (T2).

Patients will receive a baseline questionnaire (T0) by postal mail at inclusion. They will be asked to bring the completed questionnaire to the consultation. Before the consultation, patients will be asked to complete a one page questionnaire (T1). All subsequent questionnaires (i.e., T2 at one week, T3 at 3 months and T4 at 6 months post-consultation) will be sent by postal mail, including return envelopes. Reminders are sent once, if a questionnaire has not been returned within 2 weeks. Clinical data will be collected from patients’ medical files by a research associate or nurse.

### Randomization

#### Oncologists (in training)

Oncologists are randomized to receive training or continue their standard practice. Randomization stratifies for working experience (staff versus oncologist in training). Randomization occurs in blocks of 2. To prevent predictable allocation, oncologists are allocated in sets of at least two ‘of a kind’ (either staff or in training). Randomization occurs per hospital to make sure that in each hospital approximately half of the participating oncologists is trained. Randomization lists are created by an independent methodologist. Randomization is performed by an independent researcher.

#### Patients

Patients are randomized to receive the communication aid or ‘usual care’. Randomization is performed in ALEA software for randomization in clinical trials. Randomization stratifies for three factors: (1) the condition of the treating oncologist (trained/untrained) and (2) working experience of the treating oncologist (staff/in training) and (3) the type of consultation (initial treatment planning consultation/evaluative consultation), to ensure an equal ratio in all study arms. Stratification occurs through minimization. Stratification factor 1 (trained or untrained oncologist) receives a weight of 3; factors 2 and 3 a weight of 1. A biased coin of 5 starts to work in case of a contrast of >3 between the two conditions. Randomization data in ALEA are adjusted in three specific scenarios: (1) to change the status and experience of the treating oncologist in case patients switch oncologists after randomization, (2) exclusion of a randomized patient in case the patient does not provide final written informed consent (see Procedure) and (3) exclusion of a randomized patient if only during the consultation it turns out the patient does not meet the eligibility criteria (see Participants). These adjustments will be performed and documented by an independent methodologist.

### Blinding

*Oncologists* are not blinded for their own condition. Although they are blinded for their patients’ condition, the communication aid may come up in the consultation. They will be asked whether they know or suspect the patient to have received the aid in the questionnaire (T2) right after the consultation. *Patients* are obviously not blinded for whether they received the aid or not. They are blinded for their oncologist’s condition though. *Outcomes assessors* are blinded for the condition of the oncologist. The assessors will not be informed about the patients’ condition either, yet the aid may come up in the consultation.

### Interventions

#### Oncologist training

The training is based on a recent model of SDM [6] which integrates earlier models and distinguishes four essential steps within a SDM consultation: (1) set the SDM agenda, (2) inform about the options, (3) explore patients’ values and support preference construction, (4) make or defer a decision in agreement. The training aims to address knowledge (i.e., of definition, rationale, effect and steps of SDM), attitude (i.e., awareness of preference-sensitive decisions and personal barriers as well as motivation) and skills (i.e., ability to apply the four steps in a consultation using high quality communication skills). The training is provided in small groups (3–6 participants) by an experienced trainer (i.e., a medical psychologist with ample experience in skills training for health professionals) in two sessions of both 3.5 h. Staff members and oncologists in training are trained separately to allow for a safe training environment and a match in experience and skill level. The training adopts techniques known from behavior change theories [69], such as instruction (in a reader and face-to-face), modelling (tailor made video’s illustrating SDM with a patient with advanced cancer) and exercise (role play with professional actors). Moreover, the training explicitly addresses the transfer from skills in a simulated setting to a clinical setting. In a booster session of 1–1.5 h 6 weeks post-training, participants receive individual face-to-face feedback on a videotaped consultation with a real patient. Moreover, all participants receive a consultation room tool: a pocket-size card presenting the four SDM steps with example phrases to serve as a reminder and to support participants in taking the learned skills into practice. The training was piloted with 5 oncologists in training from two hospitals and was well evaluated, with a mean satisfaction score of 8 on a scale of 10. The training was accredited by the Netherlands Association of Internal Medicine (12 CME credits).

#### Patient communication aid

The patient communication aid is a brochure with three sections: (1) patient education about SDM, (2) Question Prompt Lists and (3) a Value Clarification Exercise. The brochure first explains that, in case of metastatic or inoperable cancer, treatment decisions depend on patient preferences. A rough distinction between two options is presented, i.e. the choice between best supportive care alone or best supportive care complemented with disease-targeted treatment, such as chemotherapy. It is mentioned that for some patients there are additional options, such as experimental treatment or postponing the start of disease-targeted treatment. In the second section, patients are presented with example questions they may wish to pose in the consultation with the oncologist. These questions are designed to allow patients to receive the information required to make an informed choice. It includes questions about topics patients may find difficult to ask, such as questions about life expectancy or foregoing disease-targeted treatment. The questions are ordered on two levels: (1) the decisional situation (i.e., *start* of new treatment or *evaluation* of current treatment) and (2) for each of these situations the available options (*start*: best supportive care, disease-targeted treatment, experimental treatment, postponing; *evaluation*: continuing treatment, dose reduction, stop/change treatment). The third section consists of a Value Clarification Exercise. Patients are invited to reflect on their values by three open-ended questions about what they find important for the upcoming period and what treatment outcomes they would or would not appreciate. These questions are supported by example answers and by four diverse narratives of simulated patients. Lastly, patients are invited to complete four scaling items forcing a choice between values (e.g., take every change on a longer life versus accepting the possibility of only a short time left). It is stressed that these questions are meant to help patients elaborate on their values and preferences. A decision is made together with the oncologist.

The tool was developed in three phases. The first version was developed based on examples from the literature [40, 51, 70, 71] as well as the consultation of experts from multiple disciplines (e.g., general practice, oncology, psychology, nursing, spiritual care and hospice care). This version was presented to patients (*n* = 13), their companions (*n* = 8) and bereaved relatives (*n* = 14) in semi-structured (focus group) interviews. Based on respondents’ feedback, a second version was created, which was piloted among 18 patients in two hospitals. Patients were asked to use the aid and were surveyed and interviewed about their evaluation. On the basis of their feedback, the final aid was designed.

### Outcomes

#### Primary outcome

The primary outcome is the level of SDM as observed in the audio-recorded consultations in clinical practice, assessed with two instruments. One is the widely used Observing Patient Involvement (OPTION) tool [41, 62, 63], a 12-item coding system of physician communicative behavior associated with SDM (see Additional File 1). Items are rated by observers on a 0–4 scale and scores are transformed to give a total out of 100. Next to the general manual, a study-specific manual will be developed. Besides the OPTION12, SDM will be assessed with the 4SDM (see Additional File 1), an instrument that is newly developed and based on the 4-stage SDM model [6]. In contrast to other SDM instruments, the 4SDM (1) only assesses essential elements of SDM, leaving out all non-specific communication skills, (2) assesses SDM, irrespective of who shows the behavior, thereby giving credit to both clinicians’ and patients’ initiatives, and (3) allows for the distinction between the four SDM steps. The 4SDM has eight items (two for each step) which are coded on a 4 point scale (0–3). The 4SDM has a general as well as a study-specific manual. In an unpublished pilot study the correlation between OPTION12 and an early version of the 4SDM was high (Spearman *r* = 0.92, *n* = 50).

Two raters blinded for the condition of the oncologists and patients, will code the consultations with the OPTION12 and the 4SDM. Consultations will be double coded; each consultation will be rated on the OPTION12 by one coder and on the 4SDM by a second coder. Before the start of coding, the two raters will be trained and calibrated. Interrater agreement (IRR) will be monitored during this process. Raters will first read the general and study specific manuals and discuss their questions with the PI. Then, the raters will independently code 2 × 3 encounters that were audio-recorded in a previous observational study in the same setting [20], using all instruments. Scores will be compared and inconsistencies will be discussed to arrive at a common interpretation of the items of both instruments. The raters will be assisted in these discussions by the principle investigator (PI). Then they will double code 10 consultations from the previous observational study: 5 consultations with the OPTION12 and 5 consultations with the 4SDM. For each set of 5, IRR will be calculated. IRR will be considered sufficient if the ICC’s and the average weighted kappa’s across items are higher than .60 (substantial agreement) [72], and the raters do not differ more than one point in their scores across items and consultations. As the number of coded consultations is low and distribution of categories skewed, many row and column totals in the matrices will contain zeros. In these cases, and in these cases only, kappa’s will be prevalence-adjusted by balancing the matrix [73]. When IRR is insufficient, scores of problematic items will be discussed. This process will be repeated until sufficient IRR is achieved. Then, raters will start coding the data collected in the CHOICE trial independently. They will alternate both instruments (5 with OPTION12, then 5 with 4SDM, then 5 with OPTION12, etc). Consultations will be randomly assigned.

#### Secondary outcomes

Table 2 shows all patient/oncologist-reported outcomes, including the instruments used and the timing of assessments.

**Table 2:** Secondary outcomes self-reported by patient or oncologist

Concept	Instrument	Patient					Oncologist		
		T0	T1	T2	T3	T4	T0	T1	T2
Perceived communication efficacy	Purpose designed		x						
Evaluation of communication and decision making in consultation									
Satisfaction with communication	PSQ with one extra item on SDM			x			x^a^	x^a^	x
Perceived oncologist empathy	Purpose designed items			x					
	Purpose designed VAS			x					x
Preferred/perceived decisional role	CPS		x	x					
Patient-reported SDM	SDMQ-9			x					
Decision evaluation	DCS			x					
Trust in the oncologist	Two items from TiOS			x					
Distal outcomes									
Attitudes towards striving for quantity (length) or quality of life	QQQ			x	x	x			
Quality of life	EORTC-QLQ C30	x			x	x			
Potentially adverse outcomes									
Anxiety	STAI	x		x	x	x			
	+ 1-item VAS scale	x	x	x	x	x			
Fighting spirit	Mini-MAC subscale	x		x	x	x			
Helplessness/Hopelessness	MAC subscale	x		x	x	x			
Use and evaluation of communication aid									
Use of the communication aid	Purpose designed				x^b^	x^b^			
Evaluation of the communication aid	Purpose designed			x^b^	x^b^	x^b^			

PSQ Patient Satisfaction Questionnaire, CPS Control Preferences Scale, SDMQ-9 Shared Decision Making Queationnaire −9 items, DCS Decisional Confict Scale, TiOS Trust in Oncologist Scale, QQQ Quantity Quality Questionnaire, EORTC-QoL-C30 European Organization of Research and Treatment of Cancer Quality of Life Questionnaire, STAI Spielberger State and Trait Anxiety Inventory, MINI-MAC Mini Mental Adjustment to Cancer scale, MAC Mental Adjustment to Cancer scale. ^a^ Satisfaction with communication in simulated encounter. ^b^ For patients randomized to receive the communication aid only

A. Immediate outcomes related to communication and decision making

- To assess observed SDM in a simulated setting (before and after training), oncologists will engage in standardized patient assessments both before and after training in the intervention group and on two occasions over a period of 12–16 weeks in the control group. Two standardized patients will be trained with the use of script. These scripts reflect a patient with metastatic gastric cancer (T0) and a patient with metastatic esophageal cancer (T1) who meet with the oncologist to discuss the start of first-line palliative chemotherapy. Two experienced standardized male patients will be educated about SDM, and instructed to act in a standard way and to be rather passive and not overly emotional during the encounter. They will be taught to ask a set of standard questions in all consultations, and to apply a limited set of ‘if ….then’ rules, e.g., certain questions to ask only in case the oncologists present a choice between chemotherapy and best supportive care. They will be instructed about their preference (and the underlying values and reasoning) in case a choice was presented to them, which in both cases was to prefer chemotherapy. Oncologists will receive a simulated medical file prior to the consultation, containing the standard medical information available to them prior to first patient contact. Both the script and the medical file were developed in a multidisciplinary team including psychologists and a medical oncologist, and were adjusted on the basis of experience in the pilot study (see Interventions, Oncologist training). Oncologists will be asked about their opinion on how realistic the simulated consultation was with 2 items with Likert scale responses (1–10) specifically designed for this study. The level of SDM in the simulated consultations will be assessed with the same instruments as described above.
- Observed general communication skills will be assessed in both simulated encounters and in actual encounters in clinical practice. Two items are to be rated by the two coders (double coding for all consultations): one assessing the quality of information giving (using skills like inviting questions, structuring information, summarizing) and one assessing the quality of responding to or anticipating patient’s emotional responses (using skills like showing empathy, silences, reflecting on emotions). Both items are rated on a 5-point scale ranging from 0 ‘not or hardly visible’ to 4 ‘very frequently’, and a score of 2 representing ‘sufficient’.
- Patients’ perceived efficacy in communication will be assessed with four items measuring perceived efficacy in questions asking and communicating values and preferences prior to the consultation. The items were purposefully designed for this study. Patients provide answers on VAS scale.
- To assess patient and oncologist satisfaction with communication in the consultation we will use the 5-item Patient Satisfaction Questionnaire (PSQ) [74], with a modified version for oncologists [75]. In both versions, an item about satisfaction with patient involvement in decision making was added.
- Patient-reported empathy in the consultation (post-consultation) will be assessed with one item asking about overall perceived empathy on a VAS scale as well as an adapted 5-item version of the empathy scale used in earlier research [76] with 5 response categories. Oncologist perceived empathy will be assessed with the same 1-item VAS scale as used for patients.
- To assess congruence between preferred and perceived role in decision making, patients’ preferred (before consultation) and perceived role (after consultation) will be assessed with the widely used one-item Control Preferences Scale (CPS) [77]
- Patient-reported shared decision making will be assessed with the validated 9-item Shared Decision Making Questionnaire (SDMQ-9) [78, 79].
- Patients’ decision evaluation will be assessed with the 16-item Decisional Conflict Scale [80, 81], a widely used scale to assess patient’s uncertainty about medical decisions. The formulation of the last three items is slightly adapted (‘my’ decision into ‘this’ decision).
- Patients’ trust in the oncologist (after consultation) will be assessed with the two overall items of the Trust In the Oncologist Scale [82, 83].

B. Distal outcomes:

- The treatment decision made will be registered from the medical record and categorized (starting, foregoing, (dis)continuing, adjusting palliative systemic treatment).
- Patients’ attitudes towards striving for quantity (length) or quality of life will be assessed with the 8-item Quality Quantity Questionnaire (QQQ) [84]
- Patients’ quality of life will be assessed with the 30-item EORTC Quality of Life Questionnaire (EORTC-QLQ C30) [85], a health-related quality of life questionnaire which is specifically designed and validated for cancer patients.

C. Potentially adverse outcomes:

- Anxiety will be assessed with the validated 6-item short version of the state scale of the Spielberger State and Trait Anxiety Inventory [86], as well as a 1 item VAS scale [87]. The pre-consultation (waiting room) questionnaire (T1) will only contain the 1-item VAS scale.
- Fighting spirit will be assessed with the 4-item subscale of the Mini Mental Adjustment to Cancer (MAC) scale [88]. Of note, a loss of fighting spirit is labelled as an adverse outcome, yet it could similarly be argued that accepting instead of fighting the disease is a more adaptive coping response on the long term, particularly in case of advanced cancer.
- Helplessness/hopelessness, will be assessed with the 6-item subscale of the original Mental Adjustment to Cancer (MAC) scale [89, 90].

D. Consultation duration and patients’ use and evaluation of the communication aid

Consultation time will be registered on the basis of the audiotape. Patients’ use of the communication aid will be monitored by asking if and how patients have used the tool since the recorded consultation (T3) or in the past three months (T4). Furthermore, patients’ evaluation of the aid will be assessed with 8 items developed for the current study about the perceived usefulness of the tool in e.g., asking questions and making decisions.

#### Background characteristics

Patients will be asked for their date of birth (age) by telephone. In the patient baseline survey (T0), their personal living situation, number of children (less than 18 years old), religion, nationality, educational level and preference for information with 1 item [91] will be assessed. In the oncologist baseline survey (T0; after the first standardized patient assessment), oncologists will be asked for their date of birth, years of experience, number of palliative cancer patients they see per month, communication training experience, their role preference in decision making (their patients’ preference and their own preference with the CPS [92]) and personal death attitude (3 selected items from the Death Attitude Profile-Revised [93]).

### Statistical methods

#### Primary outcome

To account for the hierarchical nature of the data, i.e. patients nested within oncologists, multilevel regression analysis will be used to test the effect of the single interventions on the observed level of SDM in the audio-recorded consultation. Analysis will be conducted separately for the OPTION12 and the 4SDM. Random intercept and slope models will be examined. The effect of the oncologist training (level of oncologists) will be established among patients who did not receive a communication aid. The effect of the communication aid (level of patients) will be established among patients of untrained oncologists. Effects with two-sided *p*-values < .05 will be considered significant; 95CI’s as well as effect sizes will be provided. As the OPTION12 is currently the most widely used instrument to assess SDM, an intervention will be considered effective if it had a significant effect on OPTION12 scores. Baseline differences across arms on background characteristics (patient age, gender, educational level) as well as patients’ baseline measures will be examined with the appropriate statistical tests (ANOVA, Chi-squared tests). All analyses of effectiveness will be controlled for differences between arms at baseline as well as the two stratification variables (working status oncologist, type of consultation). All analyses will be intention-to-treat, i.e., all eligible consultations will be included in the analysis (irrespective of whether the communication aid was actually received or read; or whether the oncologist received the full training package).

The hypothesized additional effect of the combined intervention will most likely be smaller than the effects of the single interventions (RQ1). That is, combining the two is hypothesized to increase but not to double the effect of the single interventions. The combination of the training and the aid will be considered more effective than either or both single interventions if (1) the effect size of the combination compared to care as usual is statistically significant and (2) is at least 150% of the effect size of either or both single interventions. The study is not powered to statistically test the difference between the combination and the single interventions.

#### Secondary outcomes

The effect of the interventions on the secondary outcomes will be analyzed in multi-level regression analysis with either 2 or 3 levels, i.e., outcome assessment at different time points (level 1; if applicable), nested within patients (level 2) nested within oncologists (level 3). The effect of the training on the observed level of SDM and general communication skills in simulated patient encounters will be assessed by using a repeated measure ANOVA with time (within subjects, effect of time), condition (between subjects, effect of condition) and time x condition (effect of the training) as independent variables. To present patients’ use and evaluation of the communication aid, the appropriate descriptive statistics will be used (medians, percentages).

## Discussion

Systemic treatment for advanced cancer offers uncertain and sometimes little benefit while the burden can be high. Hence, treatment decisions require Shared Decision Making (SDM). The CHOICE trial examines both the separate as well as the combined effect of an oncologist training and a patient communication aid on SDM regarding palliative systemic treatment.

The trial has several strengths. To our knowledge, it is the first study to test both the separate and combined effect of a patient- and a physician-targeted intervention on SDM about cancer treatment in the palliative setting. A strength of the study is that both the training and the communication aid are tailored for use in initial as well as evaluative consultations about palliative systemic treatment. The far majority of studies on SDM focus on single initial treatment planning consultations. Yet, decision making in the palliative setting is characterized by many and changeable options that do not result in closure. [94] CHOICE prepares both patients and oncologists for different moments of decision making. A strength of the small-group training is that it targets oncologists’ knowledge, attitude and skills by combining many different behavior change techniques [95], such as modelling, education, discussion, role play, a booster and a pocketsize reminder card. A strength of the patient combination aid is that it creates choice awareness among patients, it can be used flexibly regardless of the type and number of available options, and it explicitly targets the two-way flow of information within the conversation. A strength of the study is its randomized controlled trial design, with concealed and computer-generated allocation and blind outcome assessment to prevent bias. Several reviews have called for more robust studies with a low risk of bias to examine the effect of communication interventions [30, 96]. Moreover, the study allows for conclusions about the effect on immediate outcomes, i.e., observed communicative behavior in simulated and actual practice, as well as more distal outcomes, such as the decisions made and patients’ quality of life.

The trial has limitations as well. First of all, it has a pragmatic design [97–99] in which the control condition concerns care as usual instead of a placebo control. Patients and oncologists are not blind for their own condition; and it is likely oncologists are not blind for their patient’s condition either. Knowing one received the intervention of interest may raise expectations and change behavior. Furthermore, we did not adopt cluster-randomization at the level of departments, yet instead randomized individual doctors and patients. This has the risk of contamination, i.e., trained oncologists could influence untrained colleagues and the interaction with patients with a communication aid could influence interactions with patients without the aid in later consultations. Lastly, although we developed a training protocol which is transferable, we cannot rule out that the effect of the training depends on the trainer’s experience, skills and personality.

The findings will be submitted for publication in peer-reviewed scientific journals and will be disseminated at national and international conferences. The results will enable evidence-based choices regarding the investment in SDM interventions targeting either oncologists, patients or both in the advanced cancer setting. Moreover, if proven effective, two evidence-based interventions are available to support oncologists and patients in making decisions about treatment for advanced cancer. Ultimately, the implementation of SDM in the care for advanced cancer patients may enhance the quality of medical decision making, and hence the quality of patients’ (end of) life.

## Additional file

Additional file 1: Instruments main outcome measures (OPTION12 and 4SDM). Instruments used to assess the main outcome (observed shared decision making). (PDF 170 kb)

## Abbreviations

4SDM: For measuring Shared Decision Making (instrument)
ANOVA: Analysis of variance
CHOICE: Choosing treatment together in cancer at the end of life (study acronym)
CME: Continuing Medical Education
CONSORT: Consolidated Standards of Reporting Trials
CPS: Control Preferences Scale (instrument)
CT/PET-CT: Computed Tomography/ Positron emission tomography–computed tomography
DA: Decision Aid
DCS: Decisional Confict Scale (instrument)
EORTC-QLQ C30: European Organization for Research and Treatment of Cancer Quality of Life Questionnaire core set (instrument)
ICC: Intraclass Correlation
MAC: Mental Adjustment to Cancer scale (instrument)
Mini-MAC: Mini Mental Adjustment to Cancer scale
OPTION12: Observing Patient Involvement tool (instrument)
PSQ: Patient Satisfaction Questionnaire (instrument)
QPL: Question Prompt List
QQQ: Quality Quantity Questionnaire (instrument)
RCT: Randomized Controlled Trial
SDM: Shared Decision Making
SDMQ-9: Shared Decision Making Questionnaire (instrument)
STAI: Spielberger State and Trait Anxiety Inventory
TiOS: Trust in Oncologist Scale
VAS: Visual Analogue Scale
