# Supplementary material for: A randomized controlled trial of a skills training for oncologists and a communication aid for patients to stimulate shared decision making about palliative systemic treatment (CHOICE): study protocol
Source: BMC Cancer. 2018 Jan 8;18:55. doi: 10.1186/s12885-017-3838-8 (PMC5759304; doi:10.1186/s12885-017-3838-8)
Supplement: Additional file 1: — Instruments main outcome measures (OPTION12 and 4SDM). Instruments used to assess the main outcome (observed shared decision making). (PDF 170 kb) [file 12885_2017_3838_MOESM1_ESM.pdf]

Additional file 1. Instruments main outcome measures (OPTION12 and 4SDM)

English Version

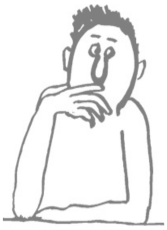

OPTION Observing patient involvement

© June 2004 glynelwyn@gmail.com

**Rater Name**		**Clinician Code**		**Date of rating**	**DD**	**MM**	**YY**	
				**Consultation number**				
				**Consultation duration** (m, s)				
				**Practitioner** (M = 1, F = 2)	**Age**		**Sex**	
				**Patient** (M = 1, F = 2)	**Age**		**Sex**	
				**New** Consultation		1		
				**Review** Consultation		2		
				**Composite** Consultation		3		
**Description of index problem**								

1	The clinician *draws attention to* an identified problem as one that requires a decision making process.	0	1	2	3	4
2	The clinician *states* that there is more than one way to deal with the identified problem ('equipoise').	0	1	2	3	4
3	The clinician *assesses* the patient's preferred approach to receiving information to assist decision making (e.g. discussion, reading printed material, assessing graphical data, using videotapes or other media).	0	1	2	3	4
4	The clinician *lists* 'options', which can include the choice of 'no action'.	0	1	2	3	4
5	The clinician *explains* the pros and cons of options to the patient (taking 'no action' is an option).	0	1	2	3	4
6	The clinician explores the patient's *expectations* (or ideas) about how the problem(s) are to be managed.	0	1	2	3	4
7	The clinician explores the patient's *concerns* (fears) about how problem(s) are to be managed.	0	1	2	3	4
8	The clinician checks that the patient has *understood* the information.	0	1	2	3	4
9	The clinician offers the patient explicit *opportunities* to ask questions during the decision making process.	0	1	2	3	4
10	The clinician elicits the patient's *preferred level of involvement* in decision-making.	0	1	2	3	4
11	The clinician indicates the need for a *decision making* (or *deferring*) stage.	0	1	2	3	4
12	The clinician indicates the need to review the decision (or *deferment)*.	0	1	2	3	4

Score	Description
0	The behaviour is not observed.
1	A minimal attempt is made to exhibit the behaviour.
2	The behaviour is observed and a minimum skill level achieved.
3	The behaviour is exhibited to a good standard.
4	The behaviour is exhibited to a very high standard.

4SDM

		No	Minimal	Sufficient	Good
**STEP 1**	**Setting the agenda**				
1	It is stated (or re-affirmed) that a decision about management or treatment needs to be made.	0	1	2	3
2	It is stated (or re-affirmed) that the decision depends on the values and preferences of the patient.	0	1	2	3
**STEP 2**	**Informing about options**				
3	The available management or treatment options are stated (or re-affirmed).	0	1	2	3
4	The pros and cons of each option are stated or re-affirmed.	0	1	2	3
**STEP 3**	**Exploring values and preference construction**				
5	The patient states the outcomes that are important to him/her (values).	0	1	2	3
6	The patient states how s(h)e appraises the (characteristics of) the management or treatment options.	0	1	2	3
**STEP 4**	**Making or deferring a decision in agreement**				
7	The patient expresses or confirms his/her preference or the (provisional) lack of a preference	0	1	2	3
8	The moment of making (or deferring) the decision is explicit and decision making occurs in agreement	0	1	2	3

September 2017

The 4SDM is not to be used in research or otherwise without the permission of the authors. The 4SDM is not to be used without the accompanying manual, which can be requested from the authors.

Contact: I.Henselmans@amc.uva.nl or e.m.smets@amc.uva.nl
